# Lifestyle Behaviors Associated With Secondary Prevention of Coronary Heart Disease Among California Adults

**Published:** 2011-02-15

**Authors:** Andrew Bellow, Joan Faith Epstein, Arti Parikh-Patel

**Affiliations:** California Department of Public Health; California Department of Public Health, Sacramento, California; California Department of Public Health, Sacramento, California

## Abstract

**Introduction:**

Heart disease is the leading cause of death in the United States. People diagnosed with coronary heart disease (CHD) are at an increased risk for illness and death. To reduce this risk, it is recommended that people who are diagnosed with CHD improve their health behaviors. The objective of this study was to assess the prevalence of associated lifestyle risk behaviors among California adults who have CHD.

**Methods:**

From 2005 through 2008, the California Behavioral Risk Factor Surveillance System collected data regarding previous diagnosis of cardiovascular diseases. We used these data to generate descriptive statistics to characterize the risk behaviors among people who had been diagnosed with CHD compared with people who had not, and developed multivariate logistic models to control for confounding variables.

**Results:**

Of total respondents, 5% reported previous diagnosis of CHD. Of respondents with CHD, three-quarters were not eating a healthful amount of fruits and vegetables, 66% were overweight or obese, 55% did not engage in regular physical activity, and nearly 15% were smokers. When we controlled for confounding variables, respondents who had been previously diagnosed with CHD were more likely than respondents who had not been diagnosed with CHD to be overweight or obese, to not exercise on a regular basis, and to be current smokers.

**Conclusion:**

Adults in California with CHD are engaging in behaviors that put them at higher risk of illness and premature death. To lower death rates due to CHD, more public health efforts should target this population.

## Introduction

Heart disease is the leading cause of death in the United States (1). An estimated 7.9 million American adults have had 1 or more myocardial infarctions (MIs) at some time in their lives, and 9.8 million have been diagnosed with angina (2). Annually, there are an estimated 610,000 new and 325,000 recurrent cases of MI and 500,000 new cases of angina (2). The combination of direct and indirect costs associated with coronary heart disease (CHD) was estimated to be $165 billion in 2009 (2). Survivors of MI are at higher risk than people who have not previously had an MI for recurrence of MI, angina, cardiac failure, stroke, and death (3). Similarly, adults diagnosed with angina have a high risk of premature death (4).

Death due to CHD has significantly decreased in the past 50 years (5,6). Half of this decline was attributed to reductions in the lifestyle risk factors that are associated with CHD (5). Guidelines based on evidence from clinical trials released by the American Heart Association and the American College of Cardiology for secondary prevention of cardiovascular diseases recommend goals for lifestyle changes. These goals include smoking cessation; blood pressure control with an emphasis on a healthful diet high in fruits, vegetables, and low-fat dairy products; regular physical activity; and weight management (7).

Lifestyle behaviors are a key aspect of prevention of illness and death among people with CHD; however, few studies have assessed people's behaviors with a population-based study (8,9). The objective of this study was to assess the prevalence of lifestyle risk behaviors associated with CHD among California adults who have CHD.

## Methods

We used cross-sectional data from the Centers for Disease Control and Prevention (CDC) Behavioral Risk Factor Surveillance System (BRFSS) from California. The BRFSS is an ongoing, cross-sectional, telephone interview survey of noninstitutionalized adults aged 18 years or older. The survey is designed to assess the prevalence and trends of health-related behaviors. The sample for California is randomly selected from 2 strata: Los Angeles County and the rest of California. The California BRFSS has been reviewed and approved annually since 1984 by the California Committee for the Protection of Human Subjects. The response rate for the survey is measured by using the proportion of eligible households that completed the interview. The response rate for the BRFSS in 2005 was 66% and decreased to 65%, 59%, and 57% in 2006, 2007, and 2008, respectively. The final sample was 29,148 adults from 2005 through 2008 (10).

Since 2005, the BRFSS has collected data regarding prior diagnosis of cardiovascular diseases. Previous diagnosis of CHD includes diagnosis of MI, angina, or other form of CHD. To determine previous diagnosis of CHD, respondents were asked, "Has a doctor, nurse, or other health professional ever told you that you had a heart attack, also called a myocardial infarction?" and "Has a doctor, nurse, or other health professional ever told you that you had angina or coronary heart disease?" Participants who responded yes for either question were included in the group for previous diagnosis of CHD. This group totaled 2,036 respondents.

The independent variables included in this analysis that could influence rates of illness and death in people with CHD were smoking, physical activity, weight, and fruit and vegetable consumption. Smoking was defined as smoking cigarettes every day or some days in the past 30 days. Irregular physical activity was defined as not engaging in moderate-intensity physical activity 5 or more times per week for 30 or more minutes at a time or vigorous-intensity physical activity 3 or more times per week for at least 20 minutes at a time. We measured unhealthy weight by calculating body mass index (BMI) using self-reported height and weight. Participants were classified into BMI categories according to the National Institutes of Health guidelines (underweight, BMI <18.5 kg/m^2^; healthy weight, BMI 18.5-24.9 kg/m^2^; overweight, BMI 25.0-29.9 kg/m^2^; obese, BMI ≥30.0 kg/m^2^) (11). People who had a BMI of at least 25.0 kg/m^2^ were considered to be at an unhealthy weight, or overweight or obese. Fruit and vegetable consumption was measured by asking about the number of times respondents ate fruits and vegetables per day during the past week. People who reported eating fruits and vegetables fewer than 5 times per day during the last 7 days were considered to have unhealthful consumption of fruits and vegetables.

We calculated descriptive statistics to compare the risk behaviors of people who had been diagnosed with CHD with those of people who had not been diagnosed with CHD. We developed separate multivariate logistic models, using each health behavior as the dependent variable. In each model, we included the following independent variables to control for confounding: age, race/ethnicity, and education level. The race/ethnicity categories were defined as non-Hispanic white, non-Hispanic black, Hispanic, and other. For each model, all health behaviors that were not the outcome of interest were included as independent variables in order to control for confounding. Certain questions on the 2008 BRFSS were asked only of a subset of the total sample. This was true of the questions on physical activity. Therefore, the total sample included in the logistic regression models was 21,048 adults. Responses were weighted by age and race/ethnicity to reflect the 2000 California population. Analyses were conducted by using SAS version 9.1 (SAS Institute, Inc, Cary, North Carolina).

## Results

Of total respondents from California, 5% reported a previous diagnosis of CHD (Table 1). More men than women reported being diagnosed with CHD, and more non-Hispanic whites reported being diagnosed with CHD than any other racial/ethnic group. Adults aged 65 or older reported the highest proportion diagnosed with CHD; adults aged 25 to 34 years reported the lowest proportion diagnosed with CHD.

Rates for current smoking were almost identical between respondents with a previous CHD diagnosis and those without (Table 2). Slightly more than half of people diagnosed with CHD did not engage in regular physical activity compared with 47% of people who had not been diagnosed with CHD. Two-thirds of people who had previously been diagnosed with CHD were overweight or obese, compared with 55% of people who had not. Of people who had previously been diagnosed with CHD, 75% ate an unhealthful amount of fruits and vegetables, compared with 77% of people who had not been diagnosed.

Results from the logistical model showed that people with CHD were 1.4 times as likely to be current smokers and 1.2 times as likely to not exercise regularly as people without a CHD diagnosis (Table 3). Respondents who had been previously diagnosed with CHD were 1.3 times as likely to be overweight or obese as people who did not have a CHD diagnosis. There were no differences in consumption of a healthful amount of fruits and vegetables between people with a CHD diagnosis and those without a CHD diagnosis.

## Discussion

This study was designed to assess the risk behaviors of adults in California who had previously been diagnosed with CHD. One of every 7 survey respondents who had been diagnosed with CHD were smokers, more than half did not engage in regular physical activity, two-thirds were overweight or obese, and more than three-quarters were not eating a healthful amount of fruits and vegetables. Compared with people not diagnosed with CHD, models indicated that people diagnosed with CHD were more likely to be smokers, to not engage in regular physical activity, and to be overweight or obese; however, there were no differences between respondents without CHD and those with CHD in terms of fruit and vegetable consumption.

Other studies that assessed these health behaviors among people with CHD in a population-based study found similar results. A study that analyzed the smoking habits of people with CHD in the United States found that smoking is high in this population (8). Another study compared fruit and vegetable consumption and physical activity levels of people in the United States with CHD with those of people without CHD. Results indicated that, when controlling for confounding variables, people with CHD were less likely to meet physical activity recommendations than people without CHD; no difference in fruit and vegetable consumption was found between the 2 groups (9). Our study differs from these studies in a few ways: we included multiple health behaviors, analyzed data for California only, and controlled for health behaviors to reduce confounding variables.

### Strengths and limitations

There were advantages to using BRFSS data. The sample size was large, and it was representative of the California population. The BRFSS solicits information on various behaviors, so we were able to allow for adjustment of these behaviors in our analysis. The BRFSS also has some inherent limitations. First, the sample is dependent on people willing to complete the survey; we have no information on characteristics of nonrespondents. However, a study using a telephone interview survey in California found that little to no difference is introduced from nonresponse bias (12). Second, people without landline telephones are not included in the sample, which means that some level of sampling bias may have occurred. Furthermore, because BRFSS data are self-reported, social desirability bias may have been introduced. Previous research has tested the reliability and validity of the BRFSS. One study found that most BRFSS questions are moderately to highly reliable and valid (13).

A strength of our study is that our results are generalizable. The BRFSS is collected as a representative sample of the state, and data were weighted to the population of California. Therefore, our results are generalizable to the state of California. Results from our study align with the results from similar studies that are representative of the US population (8,9).

There were limitations to our study design. We had no information regarding when in the participant's lifetime the CHD diagnosis occurred. Respondents may have recently been diagnosed with some form of CHD but had not had the opportunity to modify their risk behaviors. Data were not available to assess the changes in these lifestyle behaviors throughout the lives of participants to compare changes in the behaviors before and after the initial CHD diagnosis. Because of this, we could not determine if risk behaviors of people who had been diagnosed improved but were still not at the recommended level. Future research in this area could assess changes before and after diagnosis and measure the risk behaviors in a continuous fashion. Additional research could assess demographic differences in health behaviors among people with CHD.

### Conclusions

Our results show that adults in California with CHD are engaging in behaviors that put them at an increased risk of illness and death. While these behaviors may have led to respondents' CHD, results also show that adults in California with CHD are continuing to engage in unhealthy lifestyle choices after their initial diagnosis. Furthermore, they are engaging in some risk behaviors at high rates, such as smoking and irregular physical activity, and are more likely to be at an unhealthy weight than people in California who do not have CHD.

Many studies of rehabilitation for people with CHD have found that interventions significantly reduce mortality (14,15). Because no information was solicited regarding intervention or secondary prevention efforts, whether participants had previously received these benefits cannot be inferred. However, few patients who have had an MI or who have been diagnosed with angina have participated in formal outpatient cardiac rehabilitation (16-18).

Despite the decreasing number of deaths after the initial onset, incidence of MI has remained stable (5,6,19,20), which may result in an increase in the number of adults with CHD. To reduce further risk in this growing population, primary care physicians should be encouraged to recommend evidence-based cardiac rehabilitation that has been designed to specifically target the health behaviors of these patients.

## Figures and Tables

**Table 1:** Prevalence of Diagnosis of Coronary Heart Disease Among Respondents (N = 29,148)^a^ to the California Behavioral Risk Factor Surveillance System, 2005-2008

Variable	Any Form of CHD^b^	

	n	% (95% CI)
**Total**	2,036	5.0 (4.7-5.3)
**Sex**		
Male	1,027	5.8 (5.3-6.2)
Female	1,009	4.2 (3.9-4.5)
**Race/ethnicity **		
Non-Hispanic white	1,498	5.7 (5.4-6.1)
Non-Hispanic black	85	4.9 (3.7-6.2)
Hispanic	337	4.3 (3.8-4.9)
Other	116	3.5 (2.7-4.2)
**Age, y**		
18-24	22	1.5 (0.8-2.2)
25-34	54	1.4 (1.0-1.8)
35-44	102	2.0 (1.6-2.5)
45-54	208	4.0 (3.4-4.6)
55-64	411	8.3 (7.3-9.2)
≥65	1,239	16.7 (15.7-17.7)
**Education**		
8th grade or less	146	6.4 (5.1-7.7)
9th-11th grade	148	5.8 (4.6-7.1)
High school graduate	486	5.4 (4.8-6.0)
Some college	618	5.6 (5.1-6.2)
College graduate	330	3.4 (3.0-3.9)
Postgraduate degree	277	4.2 (3.6-4.9)

Abbreviations: CHD, coronary heart disease; CI, confidence interval.

a Responses were weighted by age and race/ethnicity to reflect the 2000 California population.

b Reported a previous diagnosis of myocardial infarction, angina, or CHD.

**Table 2:** Prevalence of Health Behaviors, by Diagnosis of Coronary Heart Disease, Respondents (N = 21,048)^a^ to the California Behavioral Risk Factor Surveillance System, 2005-2008

**Diagnosed With CHD^b^**	Smoker,^c^ % (95% CI)	IrregularPhysical Activity,^d^ % (95% CI)	Overweight/Obese,^e^ % (95% CI)	Unhealthful Consumption of Fruits and Vegetables,^f^ % (95% CI)
Yes	14.7 (12.6-16.9)	55.2 (51.9-58.5)	66.1 (63.4-68.8)	74.8 (72.2-77.3)
No	14.8 (14.2-15.4)	47.2 (46.3-48.1)	55.2 (54.4-56.0)	77.0 (76.3-77.6)

Abbreviations: CHD, coronary heart disease; CI, confidence interval.

a Responses were weighted by age and race/ethnicity to reflect the 2000 California population.

b Reported a previous diagnosis of myocardial infarction, angina, or CHD.

c Reported smoking cigarettes every day or some days in the past 30 days.

d Reported not engaging in moderate-intensity physical activity 5 or more times per week for 30 or more minutes at a time or vigorous-intensity physical activity 3 or more times per week for at least 20 minutes at a time.

e Body mass index ≥25.0 kg/m^2^.

f Reported eating fruits and vegetables fewer than 5 times per day in the past 7 days.

**Table 3:** Health Behaviors of Respondents (N = 21,048) With a Previous Coronary Heart Disease Diagnosis, California Behavioral Risk Factor Surveillance System, 2005-2008

**Health Behavior^a^**	Odds Ratio (95% CI)	*P* Value
Never diagnosed with any form of CHD	1 [Reference]	
Smoker^b^	1.37 (1.10-1.70)	.005
Irregular physical activity^c^	1.17 (1.01-1.36)	.04
Overweight/obese^d^	1.30 (1.11-1.52)	.001
Unhealthful consumption of fruits and vegetables^e^	0.89 (0.76-1.04)	.13

Abbreviations: CI, confidence interval; CHD, coronary heart disease.

a All models were adjusted for age, race/ethnicity, education level, and the 3 corresponding health behaviors. Responses were weighted by age and race/ethnicity to reflect the 2000 California population.

b Reported smoking cigarettes every day or some days in the past 30 days.

c Reported not engaging in moderate-intensity physical activity 5 or more times per week for 30 or more minutes at a time or vigorous-intensity physical activity 3 or more times per week for at least 20 minutes at a time.

d Body mass index ≥25.0 kg/m^2^.

e Reported eating fruits and vegetables fewer than 5 times per day in the past 7 days.
